# Comparative analysis of indocyanine green and technetium-99m for sentinel lymph node localization in breast cancer – institutional experience

**DOI:** 10.3389/fonc.2026.1817708

**Published:** 2026-05-07

**Authors:** Csaba Kósa, Hajnalka Kulcsár, Klaudia Balog, Tamás Dinya, Lóránt Illésy, Gergő Kincses, Tamás Kuna, Péter Juhász, Dezső Tóth

**Affiliations:** 1 Department of Surgery, University of Debrecen Clinical Centre, Debrecen, Hungary; 2 Institute of Pathology, University of Debrecen Clinical Centre, Debrecen, Hungary

**Keywords:** breast cancer, indocyanine green, near−infrared fluorescence, sentinel lymph node biopsy, technetium-99m

## Abstract

**Background:**

Sentinel lymph node biopsy is the gold standard for axillary staging in early breast cancer. The conventional dual-tracer technique using technetium-99m (Tc-99m) and blue dye is highly effective but limited by radiation exposure and logistical complexity. Indocyanine green (ICG) fluorescence imaging offers a radiation-free, real-time alternative. This study aimed to assess the diagnostic performance and clinical feasibility of ICG-guided SLNB compared with the standard Tc-99m-nanocolloid method.

**Methods:**

This prospective, open label, single center paired diagnostic comparison trial was conducted at the Department of Surgery, University of Debrecen Clinical Centre, Hungary. Eligible patients had biopsy-confirmed ductal carcinoma *in situ* or clinically node-negative invasive breast cancer (T1–T3) undergoing breast-conserving surgery or mastectomy. Periareolar injections of 99mTc-nanocolloid and 5 mg ICG were performed according to standardized international SLNB protocols. Lymphatic mapping was achieved with gamma-radiation detecting and near-infrared fluorescence imaging. Sentinel node detection rate, number of retrieved nodes, metastatic involvement, and adverse events were recorded and analyzed.

**Results:**

A total of 106 patients were included. ICG fluorescence achieved a sentinel node detection rate of 90,6%, compared with 97,1% for Tc-99m. A median of 2,1 lymph nodes were excised per patient. 21.7% received neoadjuvant systemic therapy and 63.2% had breast conserving surgery. The overall per-patient sentinel lymph node (SLN) detection rate was 90.6% with indocyanine green (ICG), 97.1% with technetium-99m (Tc-99m), and 98.1% with the combined technique. On a per-node basis (n = 221), detection rates were 88.2%, 83.7%, and 100%, respectively. Among patients with positive SLNs (n = 34), metastatic node detection was 94.1% using ICG, 79.4% with Tc-99m, and 100% with the combined approach. In both upfront surgery and neoadjuvant subgroups, no statistically significant differences were observed between detection methods (p > 0.05); however, interpretation of neoadjuvant results was limited by the small sample size. No ICG-related complications or adverse events were reported.

**Conclusions:**

ICG fluorescence-guided SLNB alone is a safe, effective, and radiation-free alternative to conventional radioisotope mapping. Its high detection accuracy, simplicity, and real-time visualization support its integration into standard breast cancer surgery protocols. Further multicenter studies are warranted to validate long-term oncological outcomes.

## 1 Introduction

Breast cancer remains the most frequently diagnosed malignancy among women globally, accounting for over 2,3 million new cases and approximately 665,000 deaths in 2022 (1). Accurate assessment of axillary lymph node status remains crucial for prognosis and for guiding adjuvant treatment strategies. Sentinel lymph node biopsy (SLNB) has replaced axillary lymph node dissection as the standard staging approach in clinically node-negative disease, significantly reducing the morbidity, including lymphedema, swelling of the arm and sensory loss associated with axillary clearance (2, 3).

The conventional dual-tracer technique - combining technetium-99m (Tc−99m) Sulphur colloid with blue dye - achieves high sensitivity and low false-negative rates. Although this procedure is highly effective, it has several disadvantages. These include radiation exposure for both patients and medical staff, strict regulatory requirements, limited availability of Tc-99m and the potential for allergic reactions to blue dye. Local spread of the dye may prevent complete removal of the primary tumor. Moreover, the short half-life of Tc-99m and its reliance on the Nuclear Medicine Department create logistical difficulties. Such constraints emphasize the need for accurate, radiation-free alternatives that can address these shortcomings (4). Sentimag (Sysmex) is a magnetic technique feasible for SLNB, with an identification rate that is not inferior to the standard technique (5).

Indocyanine green (ICG), a Tri carbocyanine dye that fluoresces in the near-infrared (NIR) range (peak emission approximately 830 nm), offers real-time visualization of lymphatic drainage without ionizing radiation. Introduced in breast surgery in the early 2000s, ICG has gained prominence due to its accessibility, safety profile, and ability to produce dynamic intraoperative images (6–9).

This manuscript summarizes our institutional experience comparing ICG fluorescence imaging with radioisotope mapping in the use of sentinel lymph node detection in breast cancer patients. Emphasis is placed on efficacy and potential for integration into standard practice.

## 2 Materials and methods

This was a prospective, open label, single center paired diagnostic comparison study with a non-inferiority hypothesis: that ICG fluorescence mapping is not inferior to Tc 99m mapping for SLN detection. Each enrolled patient received both tracers and served as their own control. Analyses were conducted both on a per patient (per case) basis — defined as whether at least one SLN was identified by the technique in that patient — and on a per node basis — defined by individual lymph nodes retrieved and assessed at pathology.

All patients had breast cancer surgery in the Department of Surgery, University of Debrecen Clinical Centre, Hungary. Participants met the following inclusion criteria: age over 18 years; ASA I-III. status, core biopsy-confirmed non-invasive or invasive (T1-T3) breast cancer; clinically node-negative status verified by preoperative axillary ultrasound/aspiration cytology; undergoing breast-conserving surgery or mastectomy. While ICG contains sodium iodide, patients with known allergies to iodine were excluded. Metastatic disease, presence of positive lymph nodes in other position than axilla, T4 tumors and other primary malignant disease were also exclusion criteria.

All sentinel lymph node biopsies were conducted in accordance with a standardized study protocol, consistent with international guidelines (10). As part of standard care, 99mTc-nanocolloid was administered periareolarly and subcutaneously in the tumor-bearing quadrant at the Department of Nuclear Medicine the day before surgery. A fixed dose of 100 MBq was used for all patients. The optimal dose of ICG was determined based on literature (11). Verdye^®^ (Diagnostic Green Ltd., Westmeath, Ireland) 25 mg was dissolved in 10 mL sterile, distilled water for injections (2.5 mg/mL). For each SLNB, a total of 5 mg (2 mL) dose of ICG was injected, 1 mL intradermal and 1 mL subareolar 10–15 minutes before surgery, followed by gentle manual massage at the edge of the areola at either the 10 o’clock (right breast) or 2 o’clock (left breast) position over the injection site to facilitate lymphatic uptake. For dynamic mapping of lymphatic vessels and sentinel nodes approved near-infrared fluorescence imaging was used (EleVision™ IR Platform, (Medtronic/DeepOptics). After injection, ICG drained through the lymphatic system towards the SLN. Before incision, percutaneous ICG projection and Technetium 99m radiation was recorded 15 minutes after ICG administration. The ICG fluorescent lymph nodes were removed first using the intraoperative near-infrared fluorescence detecting camera. ICG-marked lymph nodes were tested ex vivo for 99mTc-uptake with a handheld gamma probe (Crystal Probe SG04, Cristal Photonics). Next, the axilla was explored with the gamma-probe for possible residual 99mTc-activity. If found, residual lymph nodes were excised and sent for histological evaluation separately. Lymph nodes were saved in three separate containers depending on either the ICG, or technetium-99m uptake or both. The pathology report included number of lymph nodes examined, and how many were benign, contained isolated tumor cells (<0.2 mm), micrometastasis (0.2–2 mm) or macrometastasis (>2 mm). Information on extracapsular invasion was also reported.

Parameters analyzed included sentinel node detection rate, number of nodes retrieved, metastatic involvement of lymph nodes, and adverse events. Data were collected prospectively and analyzed retrospectively using SPSS 29. Continuous variables were expressed as mean ± standard deviation (SD), and categorical variables as percentages. Non-inferiority was defined as the lower limit of the 90% confidence interval of the sentinel lymph node detected proportion ratio (DPR; detected proportion with ICG divided by that with Tc-99m) being no less than 0.8. Said confidence interval was calculated based on a normal approximation of the sampling distribution of the difference between two binomial proportions. Statistical significance was determined using McNemar’s test or chi-square test, with p < 0.05 considered significant.

Final histopathological examination of all excised sentinel lymph nodes (separately labeled by tracer uptake) was considered the reference standard for determining true sentinel nodes and for assessing presence of metastasis.

## 3 Results

### 3.1 Patient and tumor characteristics

A total of 106 patients were included in the study. The mean age was 59.9 ± 12.2 years. Most patients were postmenopausal (82.1%), while 17.9% were premenopausal. among the 11 cases of ductal carcinoma *in situ* (dcis), the mean tumor diameter was 6.1 ± 3.4 cm, whereas for invasive carcinomas (n = 95) the mean diameter was 2.8 ± 2.5 cm. Regarding tumor stage, 10.4% were classified as Tis, 43.4% as T1, 37.7% as T2, and 8.5% as T3. The immunohistochemical subtype distribution of invasive carcinomas (n = 95) was as follows: luminal a – 44.2%, luminal b – 43.2%, her2-positive – 4.2%, and basal-like (triple-negative) – 8.4%. concerning tumor grade, grade 1 was found in 23.6%, grade 2 in 51.9%, and grade 3 in 24.5% of cases. most patients were node-negative (cn0, 93.4%) at the time of diagnosis, while 6.6% had initially node-positive disease (turned into ycn0 after neoadjuvant therapy). neoadjuvant therapy was administered to 21.7% of patients, whereas 78.3% underwent primary surgery. Regarding the type of surgery, breast-conserving procedures were performed in 63.2%, and mastectomy in 36.8% of cases. Postoperative complications occurred in 4.7% of patients, all of which were surgical (Clavien–Dindo grade ≤ II). No allergic or adverse reactions were observed (**Table 1**).

**Table 1:** Patient and tumour characteristics.

Characteristics (N=106)	n (%)
Age (years), mean +/- SD	59.9 +/-12.2
Menopausal status	
premenopausal	19 (17.9%)
postmenopausal	87 (82.1%)
DCIS diameter (cm), mean+/-SD (N=11)	6.1+/-3.4
Invasive tumour diameter (cm) mean+/-SD (N=95)	2.8+/-2.5
T-staging	
Tis	11 (10.4%)
T1	46 (43.4%)
T2	40 (37.7%)
T3	9 (8.5%)
Immunhistological type (invasive, N=95):	
Luminal A	42 (44.2%)
Luminal B	41 (43.2%)
HER2+	4 (4.2%)
Basal-like (triple negative)	8 (8.4%)
Tumour Grade	
Grade1	25 (23.6%)
Grade2	55 (51.9%)
Grade3	26 (24.5%)
Nodal status:	
cN0	99 (93.4%)
ycN0 (originally cN1)	7 (6.6%)
Neoadjuvant therapy	
Yes	23 (21.7%)
No	83 (78.3%)
Type of Surgery	
Mastectomy	39 (36.8%)
Breast conserving surgery	67 (63.2%)
Complications	5 (4.7%)
All complications were surgical, Clavien-Dindo Grade I-II.	
No allergic or adverse reactions were observed.	

### 3.2 Sentinel lymph node detection rates

221 lymph nodes were retrieved and analyzed histopathologically and were categorized by tracer uptake into three groups: ICG+/Tc 99m+ (both), ICG+/Tc 99m− (ICG only), and ICG−/Tc 99m+ (Tc 99m only). Detection rates are reported per patient (at least one SLN identified) and per node (proportion of all histologically identified sentinel nodes that were detected by each tracer). Histopathology of excised nodes was used as the reference standard for true sentinel nodes and for determining whether nodes were metastatic.

The overall sentinel lymph node (SLN) detection rate among 106 patients was 90.6% (95% CI, 83.3–95.4) using indocyanine green (ICG) and 97.1% (95% CI, 92.0–99.4) with technetium-99m (Tc-99m). When both tracers were combined, the detection rate reached 98.1% (95% CI, 93.4–99.8). Considering all sentinel lymph nodes identified at pathology (n = 221), ICG detected 195 nodes (88.2%; 95% CI, 83.2–92.2), Tc-99m identified 185 nodes (83.7%; 95% CI, 78.2–88.3), and the combination of both methods achieved a detection rate of 100% (95% CI, 98.3–100.0). For positive SLNs (n = 34), ICG detected 94.1% (95% CI, 80.3–99.3), Tc-99m detected 79.4% (95% CI, 62.1–91.3), and the combination detected 100% (95% CI, 89.7–100.0). Among micrometastases (n = 3), all cases (100%) were detected with ICG (95% CI, 29.2–100.0), whereas Tc-99m identified 66.7% (95% CI, 9.4–99.2). For macrometastases (n = 31), the detection rate was 93.5% (95% CI, 83.3–99.9) for ICG and 80.6% (95% CI, 66.3–94.5) for Tc-99m (**Table 2**).

**Table 2:** Sentinel lymph node detection rates.

Parameter	ICG		Tc-99m		ICG+Tc-99m		
	n (%)	(95% CI)	n (%)	(95% CI)	n (%)	(95% CI)	Detected proportion ratio (90%CI)
Overall sentinel lymph node detection rate (n=106)	96 (90.6%)	(83.3–95.4)	103 (97.1%)	(92.0–99.4)	104 (98.1%)	(93.4–99.8)	0.93 (0.88–0.99)
All sentinel lymph nodes found at pathology (n=221)	195 (88.2%)	(83.2–92.2)	185 (83.7%)	(78.2–88.3)	221 (100%)	(98.3–100.0)*	1.05 (0.99–1.12)
Positive sentinel lymph node detection rate (n=34)	32 (94.1%)	(80.3–99.3)	27 (79.4 (%)	(62.1–91.3)	34 (100%)	(89.7–100.0)*	1.19 (1.02–1.35)
Micrometastasis (n=3)	3 (100%)	(29.2–100.0)	2 (66.7%)	(9.4–99.2)	3 (100%)	(29.2–100.0)*	1.50 (0.83–2.17)
Macrometastasis (n=31)	29 (93.5%)	(83.3–99.9)	25 (80.6%)	(66.3–94.5)	31 (100%)	(88.8–100.0)*	1.16 (0.99–1.33)

Tc-99m indicates radioactive technetium.

Sentinel lymph node detection rates were defined as the proportion of lymph nodes detected and were presented in percentage form with exact binomial 95% confidence intervals.

*One-sided 97.5% confidence interval.

The total of 221 sentinel lymph nodes were analyses for concordance between indocyanine green (ICG) fluorescence and technetium-99m (Tc-99m) uptake. Of these, 159 nodes (71.9%) were positive for both ICG and Tc-99m (ICG+/Tc-99m+), while 36 nodes (16.3%) were positive for ICG only (ICG+/Tc-99m−). 26 lymph nodes (11.8%) were detected as Tc-99m positive and ICG negative (ICG+/Tc-99m+). Overall, the concordance rate between ICG fluorescence and Tc-99m radioisotope mapping was 83.7%, indicating a high level of agreement between the two detection methods (**Table 3**).

**Table 3:** Concordance rates of ICG and Technetium-99m in sentinel lymph node detection (n=221).

ICG status	Tc-99m+ n (%)	Tc-99m- n (%)	Total n (%)
ICG+	159 (71.9%)	36 (16.3%)	195 (88.2%)
ICG-	26 (11.8%)	0 (0%)	26 (11.8%)
Total	185 (83.7%)	36 (16.3%)	221 (100%)

ICG+ indicates lymph nodes with ICG uptake.

ICG- indicates lymph nodes with no ICG uptake.

Tc-99m+ indicates lymph nodes with technetium isotope uptake.

Tc-99m- indicates lymph nodes with no technetium isotope uptake.

### 3.3 The comparative outcome of sentinel lymph node biopsy using indocyanine green, Tc-99m, and combined technique

ICG-guided SLNB and the Tc-99m-nanocolloid method were compared in terms of nodal detection performance using McNemar’s test based on the 2×2 table of all individual lymph nodes categorized by whether or not they were detected by either method. In the entire study population (N = 106), SLNB using ICG achieved a per-case detection rate of 90.6% (96/106). The mean number of sentinel lymph nodes (SLNs) identified per patient was 2.0 ± 1.1. On a per-node basis, ICG detected 88.2% (195/221) of all sentinel nodes. Using the Tc-99m detection was successful in 97.1% of the cases (103/106) with mean number of SLNs 1.8 ± 1.0. 83.7% of nodes were detected (185/221) without reaching statistical significance (p=0.2074). Combining the two techniques led to 98.1% detection rate per cases (104/106). Among patients with histologically positive SLNs (N = 34), metastatic nodes were successfully identified using ICG in 94.1% (32/34), with Tc-99m in 79,4% (27/34) and with combination of methods in 100% (34/34) of the cases indicating a high sensitivity for nodal metastasis detection.

In the upfront surgery subgroup (N = 83), the per-case SLN detection rate using ICG was 89.2% (74/83), with Tc-99m 97.6% (81/83). The mean number of detected SLNs was lower than in the overall cohort (1.4 ± 0.6). When per-node detection rates were compared between methods, ICG identified 85.7% of nodes (144/168), whereas Tc-99m alone had a success rate of 79.2% (133/168). The combined technique achieved a detection rate of 100%. Despite this numerical difference, statistical analysis did not demonstrate a significant difference among the three methods (p = 0.1550). Similarly, the detection of positive sentinel nodes showed lower rates for Tc-99m (75.0%) compared with ICG (92.9%) and the combined technique (100%); however, this difference did not reach statistical significance (p = 0.1797), suggesting comparable oncological performance across techniques in this subgroup.

In patients who received neoadjuvant systemic therapy (N = 23), both Tc-99m and ICG-guided SLNB demonstrated a high per-case detection rate (95.7%). The mean number of identified SLNs was 2.3 ± 1.3, exceeding that observed in the upfront surgery group. Per-node detection rates were consistently high across all techniques, with ICG detecting 96.2% of nodes and Tc-99m detecting 98.1%. No statistically significant differences were observed between methods (p > 0.9999). Likewise, all positive sentinel lymph nodes were successfully identified by each technique in this subgroup, with identical detection rates and no significant inter-method differences (p > 0.9999). Of course, given the low case number, no meaningful conclusions can be drawn from this comparison (**Table 4**).

**Table 4:** The comparative outcome of sentinel lymph node biopsy (SLNB) using indocyanine green (ICG), Tc-99m, and combined technique.

Parameter	Method			P-value
	ICG	Tc-99m	ICG +Tc-99m	ICG vsTc-99m
Overall (N = 106)				
SLN detection rate (Per case), n (%)	96 (90.6%)	103 (97.1 %)	104 (98.1 %)	
Success rate of SLNB, n (%)				
Fail SLN mapping	10 (9.4%)	3 (2.8 %)	2 (1.9 %)	
Number of detected SLN (Mean ± SD)	2.0± 1.1	1.8 ± 1.0	2.1 ± 1.1	
Nodal detection rate (Per node), n (%) (N=221)	195 (88.2 %)	185 (83.7 %)	221 (100%)	0.2074
Positive SLN detection rate, n (%) (N=34)	32 (94.1 %)	27 (79.4 %)	34 (100 %)	0.1797
Upfront surgery (N = 83)				
SLN detection rate (Per case), n (%)	74 (89.2 %)	81 (97.6 %)	82 (98.8 %)	
Success rate of SLNB, n (%)				
Fail SLN mapping	9 (10.8 %)	2 (2.4 %)	1 (1.2 %)	
Number of detected SLN (Mean ± SD)	1.4 ± 0.6	1.1 ± 0.5	1.5 ± 0.7	
Nodal detection rate (Per node), n (%) (N=168)	144 (85.7 %)	133 (79.2 %)	168 (100 %)	0.1550
Positive SLN detection rate (N = 28)	26 (92.9 %)	21 (75.0 %)	28 (100 %)	0.1797
Post neoadjuvant chemotherapy (N = 23)				
SLN detection rate (Per case) n (%)	22 (95.7 %)	22 (95.7 %)	22 (95.7%)	
Success rate of SLNB, n (%)				
Fail SLN mapping	1 (4.3 %)	1 (4.3 %)	1 (4.3 %)	
Number of detected SLN, (Mean ± SD)	2.3 ± 1.3	2.4 ± 1.3	2.3 ± 1.4	
Nodal detection rate (Per node), n (%)(N = 53)	51 (96.2 %)	52 (98.1 %)	53 (100 %)	>0.9999
Positive SLN detection rate, n (%) (N=6)	6 (100 %)	6 (100 %)	6 (100 %)	>0.9999

ICG, Indocyanine green; Tc-99m, Technetium-99m; SLN, sentinel lymph node; SLNB, sentinel lymph node biopsy.

Overall, statistical analysis revealed no significant differences in sentinel node detection rates between ICG, Tc-99m, and the combined approach, either in the upfront surgery or neoadjuvant setting. These findings indicate that ICG-guided SLNB provides detection efficacy comparable to established radiotracer-based methods, supporting its reliability as an alternative or complementary technique in different clinical scenarios. No ICG-related complications or adverse events were reported.

Completion axillary dissection was not performed for patients in this cohort and long term follow up is currently limited; therefore we cannot provide an observed false negative rate or axillary recurrence rate within this manuscript. These outcomes are being collected in ongoing follow up and will be reported separately.

## 4 Discussion

The results of this study and those reported in the literature confirm the reliability of ICG-guided SLNB in early breast cancer. The study demonstrated that ICG fluorescence imaging was non-inferior to Tc-99m. The concordance rate between the two tracers was 71.9%. At least one lymph node was successfully identified in 90.6% of cases using ICG fluorescence, compared with 97.1% using Tc-99m. The ICG-technique achieved a detection rate of 94.1% for pathological lymph nodes while the isotope reached 79.4%. The median number of lymph nodes excised was 2,1. A multicenter analysis demonstrated a 99% detection rate using near-infrared fluorescence (12), while another paper reported a superior detection rate of 99% with ICG compared to 89% with blue dye (13, 14). Multiple meta-analyses have verified equivalence or superiority of ICG compared with conventional tracers (15–17).

We note that our paired design, in which each patient received both tracers and all excised nodes were histologically examined, is a within patient diagnostic comparison rather than a classical single arm non inferiority randomized trial. This paired approach strengthens internal comparison because each patient acts as their own control; however, it may limit external generalizability and we acknowledge this as a limitation.

Recent investigations have expanded the scope of ICG use confirming comparable results between ICG and Tc−99m in both upfront and post-neoadjuvant settings (18, 19).

The principal advantages of ICG fluorescence include its absence of ionizing radiation, real-time imaging capability, simplified logistics, and cost-effectiveness (19). The technique eliminates the need of additional hospital visit for nuclear medicine insertion and radiation safety protocols thereby reducing patient discomfort (7, 20). ICG has demonstrated superior safety compared with Tc-99m and blue dye, showing fewer adverse events (21, 22). Furthermore, it facilitates direct visualization of lymphatic flow and sentinel node location, improving intraoperative precision and potentially reducing false negative rates (23).

Economically, ICG fluorescence-guided SLNB is estimated to be at least five times less costly than procedures using 99mTc-nanocolloid (24) ICG itself is an inexpensive agent and the fluorescence imaging systems can be used for multiple surgical applications, making the initial investment cost-effective for hospitals (11, 25). Finally, ICG fluorescence is both patient- and surgeon-friendly. The dye is administered after induction of general anesthesia, causing no discomfort and provides real-time visual guidance without blue tissue discoloration (18, 26). Consequently, it combines the advantages of both radioisotope and dye-based methods, contributing to lower surgical morbidity (27).

To improve detection rates and reduce false negatives, some authors recommend using a dual-mapping approach rather than a single tracer (28). However, the INFLUENCE trial results provide the first randomized data supporting the safety and efficacy of ICG alone for SLNB in early breast cancer patients. Among evaluable patients (n = 97), the overall SLN identification rate was 96.9% (ICG alone = 97.9%; ICG+ RI = 100%; ICG + blue dye = 92%) (29). These results support that RI and/or blue dye. can be safely withheld as a co-tracer without compromising detection of positive nodes in primary surgical patients.

In our study, the combined use of ICG and 99mTc-nanocolloid achieved a detection rate of 98.1%, compared with 90.6% for ICG alone. However, the number of pathological lymph nodes identified was higher with ICG alone (32/34, 94.1%) than with Technetium (27/34, 79.4%). Consistent with previous studies evaluating the added value of blue dye in combination with Tc-99m, it appears unlikely that the modest increase in detection rate has meaningful clinical benefit compared to the potential drawbacks of introducing an additional tracer (30–32). Furthermore, earlier reports suggest that the use of a second tracer, such as blue dye, may offer an advantage primarily for less experienced surgeons (31, 32).

While our study focused on mapping performance and concordance, a central consideration for any alternative SLNB technique is oncological safety. Key metrics include the false negative rate (FNR), axillary recurrence, and long-term regional control and survival. Our design (paired mapping with excision of tracer positive nodes and histopathological assessment) does not directly provide an FNR in the classical sense because FNR calculation requires a reference axillary dissection or long term follow up to capture missed metastatic nodes. In this series we did not perform completion axillary dissection on patients, and follow up duration is currently limited.

Nevertheless, larger prospective and multicenter studies have evaluated oncological safety of ICG guided SLNB. Notably, multicenter series (12) and subsequent prospective trials have reported high detection rates with low axillary recurrence rates on short to medium term follow up (14, 25). Long term observational data suggest low axillary recurrence after ICG guided SLNB, but randomized data with extended follow up remain limited. A single center study using ICG and blue dye for SLN detection – excluding patients with macrometastasis in the SLN – showed axillary recurrence in only 0.9% of patients, local and distant recurrences of 1.8% and 5.5% with a median follow-up of 42 months. The findings suggest that the ICG and blue dye technique maintains a low axillary recurrence rate comparable to traditional methods (33). Hybrid tracers and combined mapping approaches have also been studied to reduce potential false negatives (12, 36). We therefore place our detection and concordance findings in this context: while our per node and per patient detection rates are broadly consistent with prior multicenter reports, definitive statements about oncological equivalence require longer follow up or trials designed specifically to assess FNR and recurrence.

We acknowledge the slightly lower per patient detection rate with ICG (90.6% vs 97.1% for Tc 99m) and emphasize that even modest decreases may have clinical implications for axillary staging. Possible explanations for failed ICG detection include limited NIR tissue penetration (nodes deep >10–20 mm), higher BMI reducing signal, medial/central tumor drainage patterns producing deeper axillary nodes, prior breast surgery altering lymphatic channels, and technical/surgeon learning factors (11, 34–37).

The variability in injection technique, dose, and timing among institutions underscores the need for standardized protocols. Access to high-quality NIR imaging systems and training also influences adoption, particularly in resource-limited centers. Time-dependent execution due to the tendency of the solution to pass to non-sentinel lymph nodes is also notable. Dispersion of the tracer after removing the first lymph nodes might prevent the correct evaluation of further sentinel lymph nodes.

Emerging innovations seek to overcome these constraints. Hybrid tracers combining ICG with technetium (99mTc-ICG) or newer fluorophores (such as IRDye800CW) enable both preoperative lymphoscintigraphy and intraoperative fluorescence (38). Artificial intelligence-assisted fluorescence mapping and augmented-reality overlays are under exploration to enhance precision and reproducibility. The usefulness of these new discoveries in breast cancer surgery remains to be seen. Multicenter randomized controlled trials are underway to evaluate cost-effectiveness and long-term oncological outcomes (39, 40).

## 5 Conclusions

ICG fluorescence-guided SLNB represents a safe, effective, and radiation-free alternative to conventional radioisotope mapping in breast cancer. Its accuracy, rapid lymphatic visualization, and favorable safety profile render it a valuable tool for surgical oncology. Our institutional experience aligns with published evidence demonstrating high detection rates and procedural efficiency.

The integration of ICG into standard SLNB protocols could reduce dependence on nuclear medicine infrastructure and improve accessibility in community hospitals. Further randomized multicenter trials with long-term follow-up are required to consolidate its position in clinical guidelines.

## Data Availability

The raw data supporting the conclusions of this article will be made available by the authors, without undue reservation.
